# A comparative study of ZnAl_2_O_4_ nanoparticles synthesized from different aluminum salts for use as fluorescence materials

**DOI:** 10.1038/srep12849

**Published:** 2015-08-04

**Authors:** Shi-Fa Wang, Guang-Zhuang Sun, Lei-Ming Fang, Li Lei, Xia Xiang, Xiao-Tao Zu

**Affiliations:** 1 School of Physical Electronics and Institute of Fundamental and Frontier Sciences, University of Electronic Science and Technology of China, Sichuan, Chengdu, 610054, China; 2 Institute of Nuclear Physics and Chemistry, China Academy of Engineering Physics, Sichuan, Mianyang, 621900, China; 3 Institute of Atomic and Molecular Physics, Sichuan University, 610065, Chengdu, China

## Abstract

Three ZnAl_2_O_4_ samples were prepared via a modified polyacrylamide gel method using a citric acid solution with different aluminum salt starting materials, including AlCl_3_∙6H_2_O, Al_2_(SO_4_)_3_∙18H_2_O, and Al(NO_3_)_3_∙9H_2_O under identical conditions. The influence of different aluminum salts on the morphologies, phase purity, and optical and fluorescence properties of the as-prepared ZnAl_2_O_4_ nanoparticles were studied. The experimental results demonstrate that the phase purity, particle size, morphology, and optical and fluorescence properties of ZnAl_2_O_4_ nanoparticles can be manipulated by the use of different aluminum salts as starting materials. The energy bandgap (Eg) values of ZnAl_2_O_4_ nanoparticles increase with a decrease in particle size. The fluorescence spectra show that a major blue emission band around 400 nm and two weaker side bands located at 410 and 445 nm are observed when the excitation wavelength is 325 nm. The ZnAl_2_O_4_ nanoparticles prepared from Al(NO_3_)_3_∙9H_2_O exhibit the largest emission intensity among the three ZnAl_2_O_4_ samples, followed in turn by the ZnAl_2_O_4_ nanoparticles prepared from Al_2_(SO_4_)_3_∙18H_2_O and AlCl_3_∙6H_2_O. These differences are attributed to combinational changes in Eg and the defect types of the ZnAl_2_O_4_ nanoparticles.

Spinel ZnAl_2_O_4_ is known to have a wide energy bandgap (Eg), high mechanical resistance, high fluorescence efficiency, high chemical and thermal stability, high photocatalytic activity, and low surface acidity, all of which make it a suitable material for a wide range of applications, including use in photoelectronic devices, catalysts, electroluminesence displays, stress imaging devices, optical coatings, and highly efficient phosphors^1^,^2^,^3^. Based on the above mentioned applications, various morphologies of spinel ZnAl_2_O_4_ have been prepared, including one-dimensional microfibers, porous structures, nanoparticles, nanorods, nanotubes, and so on^4^,^5^,^6^,^7^,^8^. It has been noted that the optical properties of these materials are strongly dependent on their morphologies and preparation methods. In particular, ZnAl_2_O_4_ nanostructures are expected to exhibit enhanced optical and fluorescence properties usually absent in bulk ZnAl_2_O_4_. Therefore, the preparation and study of the optical and fluorescence properties of ZnAl_2_O_4_ nanostructure powder is of great interest.

Spinel ZnAl_2_O_4_ semiconductors have been synthesized using a variety of different methods, such as the solid-state reaction method^9^,^10^, a self-generated template pathway^9^, the combustion synthesis route^11^, the sol–gel method^12^, a co-precipitation approach^6^, the polymeric precursor method^13^, the citrate precursor method^4^, a hydrothermal process^7^, a solvothermal approach^14^, and the microwave-hydrothermal route^15^. The particle size of ZnAl_2_O_4_ has a large effect on its optical and fluorescence properties. Generally, smaller particles have a relatively larger specific surface area, and therefore have a larger amount of dangling and unsaturated bonds on the particle surface. This in turn affects the defect levels and fluorescence properties of the powder^16^. However, the main disadvantage of preparing spinel ZnAl_2_O_4_ by the traditional synthesis routes, such as the co-precipitation approach, the solid-state reaction method, and others, is the large particle size of the product.

The polyacrylamide gel route is a very good sol-gel method for the preparation of superfine nanoparticles^17^. Appropriate selection of a chelating agent, monomer systems, initiator, pH value, and sintering temperature can significantly improve the quality of the prepared nanoparticles^17^. In addition, different aluminum salts, i.e. different anionic species in the precursor solutions, can greatly influence the morphology, phase purity, and optical and fluorescence properties of the ZnAl_2_O_4_. However, most previously reported studies have only used a single aluminum salt as a starting material and have not investigated the influences of different aluminum salts on the morphology, structure, and optical and fluorescence properties of the obtained ZnAl_2_O_4_.

In this study, three different aluminum salts are used as starting materials to synthesize three ZnAl_2_O_4_ gels via a polyacrylamide gel route, specifically aqueous solutions of citric acid with Al_2_(SO_4_)_3_∙18H_2_O, AlCl_3_∙6H_2_O, or Al(NO_3_)_3_∙9H_2_O were used under identical conditions. In order to obtain superfine nanoparticles, *N*,*N’*-methylene-bisacrylamide was used as a cross-linking agent, and glucose was used to prevent gel collapse. After sintering these xerogels, three ZnAl_2_O_4_ nanostructure samples were obtained. Their phase purity, morphologies, and optical and fluorescence properties were then characterized and compared. The objective of the present work is to investigate the influence of different aluminum salt starting materials on the resulting ZnAl_2_O_4_ nanostructures and on their optical and fluorescence properties.

## Results

**Figure 1:**
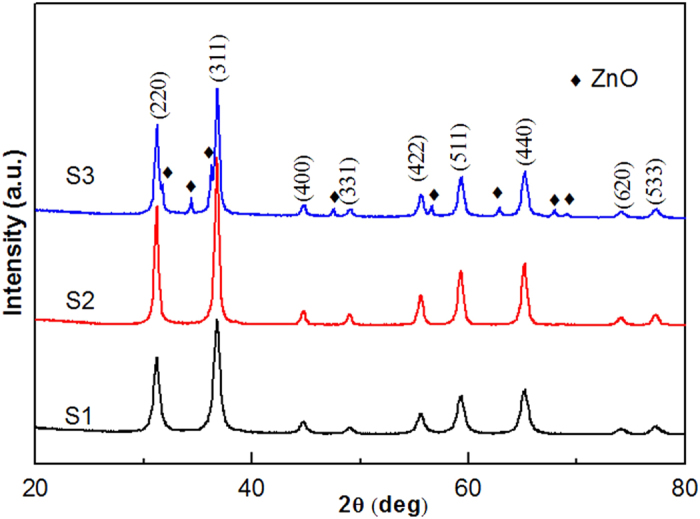
XRD patterns of ZnAl_2_O_4_ nanoparticles prepared from (S1) Al_2_(SO_4_)_3_∙18H_2_O, (S2) AlCl_3_∙6H_2_O, and (S3) Al(NO_3_)_3_∙9H_2_O and sintered at 600 °C.

The obtained ZnAl_2_O_4_ xerogels decomposed into products after being sintered at 600 °C for 5 h in air. Figure 1 shows the XRD patterns of ZnAl_2_O_4_ nanoparticles prepared from (S1) Al_2_(SO_4_)_3_∙18H_2_O, (S2) AlCl_3_∙6H_2_O, and (S3) Al(NO_3_)_3_∙9H_2_O. It can be seen that samples S1 and S2 have crystallized in a single phase with a spinel structure and with space group O*7h*, but sample S3 contains small amounts of ZnO (JCPDS card No. 36–1451) impurities in addition to the major phase of spinel ZnAl_2_O_4_ structure (JCPDS card No. 05–0669). According to the literature^2^,^18^, the relevant reactions can be described by the following equations:


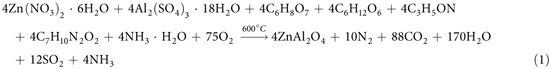



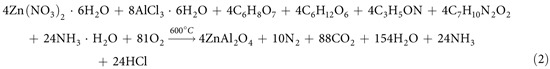



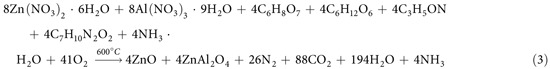


For samples S1 and S2, the observed diffraction peaks at 2*θ* are 31.22, 36.77, 44.69, 48.98, 55.52, 59.27, 65.06, 73.97, and 77.12 and can be ascribed, respectively, to the (220), (311), (400), (331), (422), (511), (440), (620), and (533) planes of ZnAl_2_O_4_. The mean grain size of samples S1, S2, and S3 were quantitatively evaluated based on the line broadening of the (220), (311), (511), and (440) peaks using the Scherrer formula, to be 13, 16, and 24 nm, respectively. XRD results indicate that the choice of the aluminum salts also has an influence on the phase purity of the final product. A possible reason for the formation of impurity phases when using citric acid as a chelating agent is that citric acid has a relatively weak coordinating capacity toward the metal ion of Al(NO_3_)_3_∙9H_2_O, and hence the formed metal complexonate is not expected to be highly stable.

**Figure 2:**
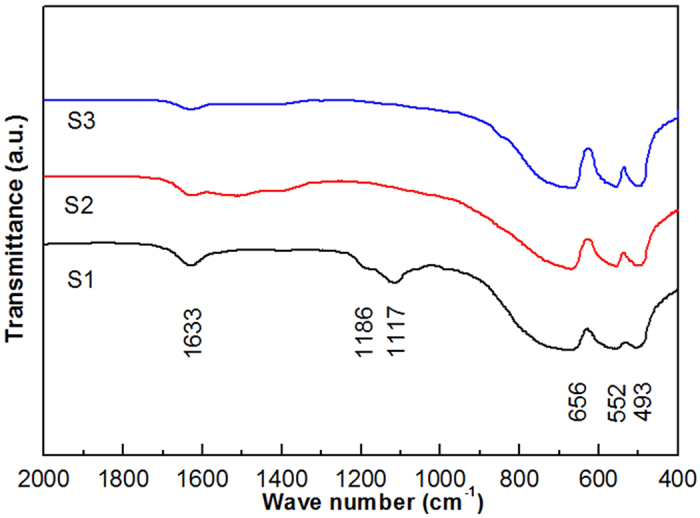
FT-IR spectrum of ZnAl_2_O_4_ nanoparticles prepared from (S1) Al_2_(SO_4_)_3_∙18H_2_O, (S2) AlCl_3_∙6H_2_O, and (S3) Al(NO_3_)_3_∙9H_2_O and sintered at 600 °C.

Fourier transform infrared (FT-IR) spectra of the ZnAl_2_O_4_ nanoparticles prepared from (S1) Al_2_(SO_4_)_3_∙18H_2_O, (S2) AlCl_3_∙6H_2_O, and (S3) Al(NO_3_)_3_∙9H_2_O are shown in Fig. 2. The FT-IR spectra show a series of absorption peaks in the range of 400–2000 cm^−1^. According to the specific frequencies of the absorption peaks, the functional groups existing in the samples can be deduced. Peaks at 1633, 656, 552, and 493 cm^−1^ are present in all samples, and are assigned to the H-O-H bending vibration of adsorbed water^19^, Al-O symmetric stretching vibration (ν_1_)^19^,^20^,^21^,^22^, Al-O symmetric bending vibration (ν_2_)^19^,^20^,^21^,^22^, and Al-O asymmetric stretching vibration (ν_3_)^21^,^22^, respectively. For sample S2, the peaks located at 1186 and 1117 cm^−1^ are attributed to the S=O asymmetric stretching vibration^23^ and the S-O symmetric stretching vibration^23^,^24^, respectively.

**Figure 3:**
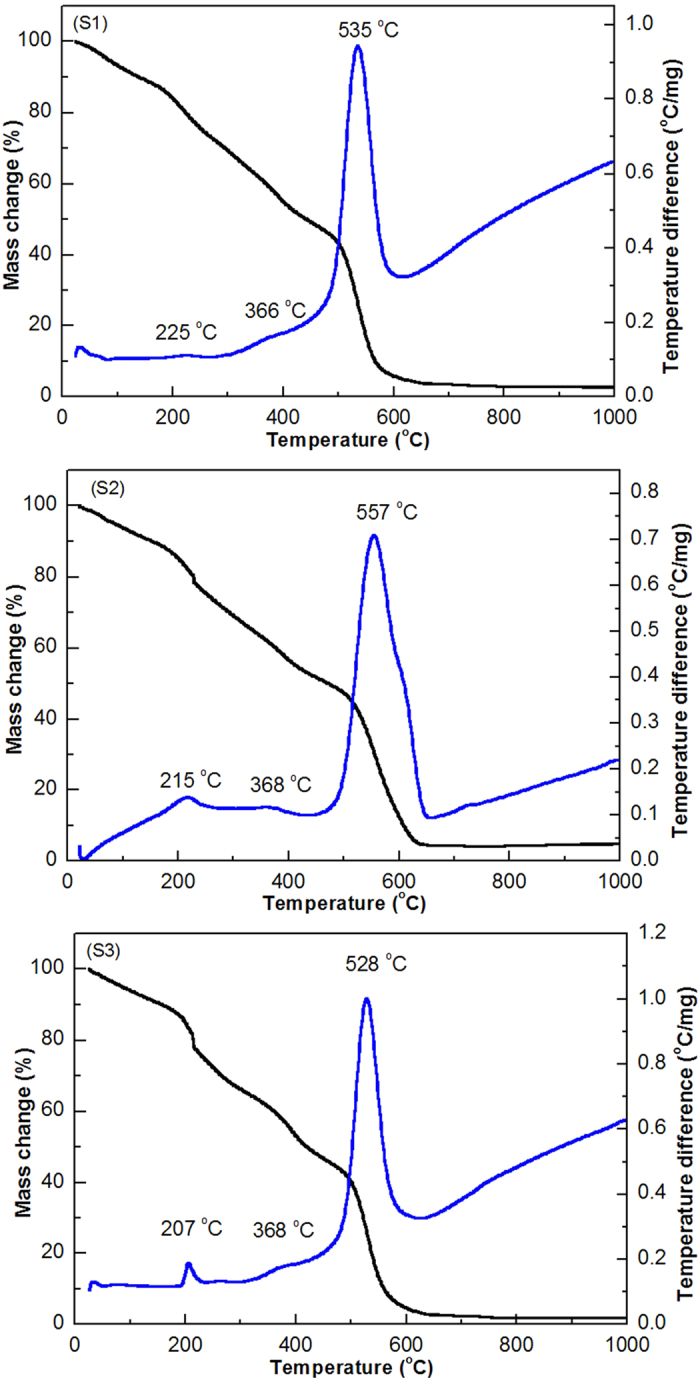
TG/DTA curves of ZnAl_2_O_4_ xerogel prepared from (S1)Al_2_(SO_4_)_3_∙18H_2_O, (S2) AlCl_3_∙6H_2_O, and (S3) Al(NO_3_)_3_∙9H_2_O.

Figure 3 shows the TG/DTA curves of the ZnAl_2_O_4_ xerogels obtained from (S1) Al_2_(SO_4_)_3_∙18H_2_O, (S2) AlCl_3_∙6H_2_O, and (S3) Al(NO_3_)_3_∙9H_2_O. There are four weight loss stages observed for each sample. The first weight loss stage is seen at a low temperature range (before 200 °C) and corresponds to the evaporation of surface water in the ZnAl_2_O_4_ xerogel precursors^25^,^26^. The second weight loss stage (around 200–250 °C) is due to the evaporation of structural water^25^,^26^. The third weight loss stage (between 250–400 °C) is due to the decomposition of small molecular organic compounds. The largest and final weight loss stage (around 400–620 °C) is due to decomposition of complexes, glucose, and the polyacrylamide side-chain, as well as combustion of the polyacrylamide backbone and other residues^27^,^28^. The total weight loss measured for the ZnAl_2_O_4_ xerogel precursors were 97.31% for (S1) Al_2_(SO_4_)_3_∙18H_2_O, 95.201% for (S2) AlCl_3_∙6H_2_O, and 98.234% for (S3) Al(NO_3_)_3_∙9H_2_O. In Fig. 3 (S1), the main endothermic peak appeared at around 535 °C and corresponds to the thermal decomposition of the complexes, polyacrylamide backbone, and other residues originating from Al_2_(SO_4_)_3_ ∙18H_2_O. Meanwhile, the main endothermic peaks appeared at 557 °C (Fig. 3(S2)) for the ZnAl_2_O_4_ xerogel precursor obtained from AlCl_3_∙6H_2_O, and at 528 °C (Fig. 3(S3)) for (S3) Al(NO_3_)_3_∙9H_2_O. The slightly different decomposition temperatures of the three ZnAl_2_O_4_ xerogels may be related to differences in the microstructures caused by the presence of different anionic species (Cl^−^, SO_4_^2−^, and NO_3_^−^) in the synthesis process. The chemical reaction is complete at ~ 600/620 °C and results in the formation of ZnAl_2_O_4_ nanoparticles. However, for ZnAl_2_O_4_ nanoparticles prepared from Al_2_(SO_4_)_3_∙18H_2_O, a higher heat treatment temperature is usually needed to improve the phase purity.

**Figure 4:**
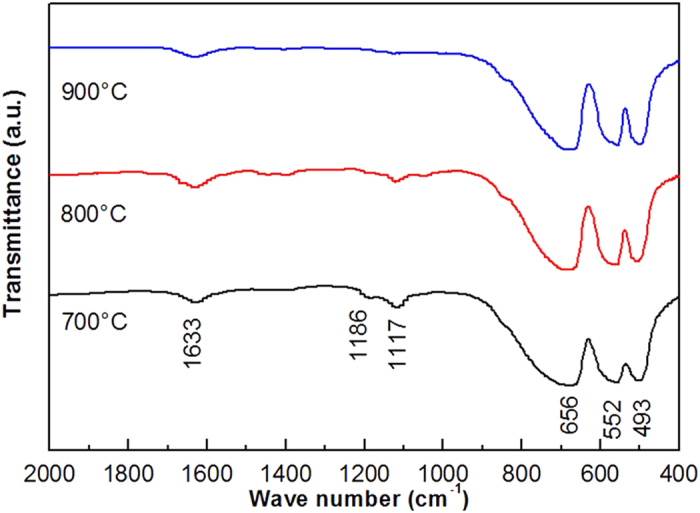
FT-IR spectrum of ZnAl_2_O_4_ xerogel prepared from Al_2_(SO_4_)_3_∙18H_2_O and sintered at 700, 800, and 900 °C.

To confirm whether the formation of ZnAl_2_O_4_ nanoparticles prepared from Al_2_(SO_4_)_3_∙18H_2_O needed a higher heat treatment temperature, FT-IR measurements were carried out using a Bruker IFS 66 v/S spectrometer. The FT-IR spectra of the ZnAl_2_O_4_ xerogel prepared form Al_2_(SO_4_)_3_∙18H_2_O and sintered at different temperatures are presented in Fig. 4. Here is can be seen that the S=O asymmetric stretching vibration (1186 cm^−1^) and S-O symmetric stretching vibration (1117 cm^−1^) peak intensities decrease with the increase of sintering temperature. This result indicates that the SO_4_^2 −^ anion coordinates to Zn and Al cations and forms a bridged bidentate structure^23^,^29^,^30^.

In the case of the sample obtained by sintering the xerogel at 900 °C, all of the organic peaks disappear except for the H−O−H peak (1633 cm ^−1^). These results indicate that the effects of aluminum salts and sintering temperature on the phase purity of ZnAl_2_O_4_ cannot be neglected. Based on the subtle information gathered from the XRD and FT-IR results, the relevant reactions can be described as follows:


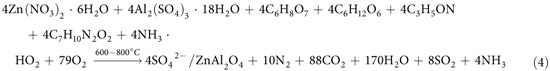



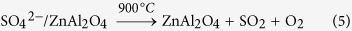


The results indicate that the reaction (1) cannot occur at 600 °C, and that a higher sintering temperature is needed for the formation of pure ZnAl_2_O_4_ nanoparticles.

**Figure 5:**
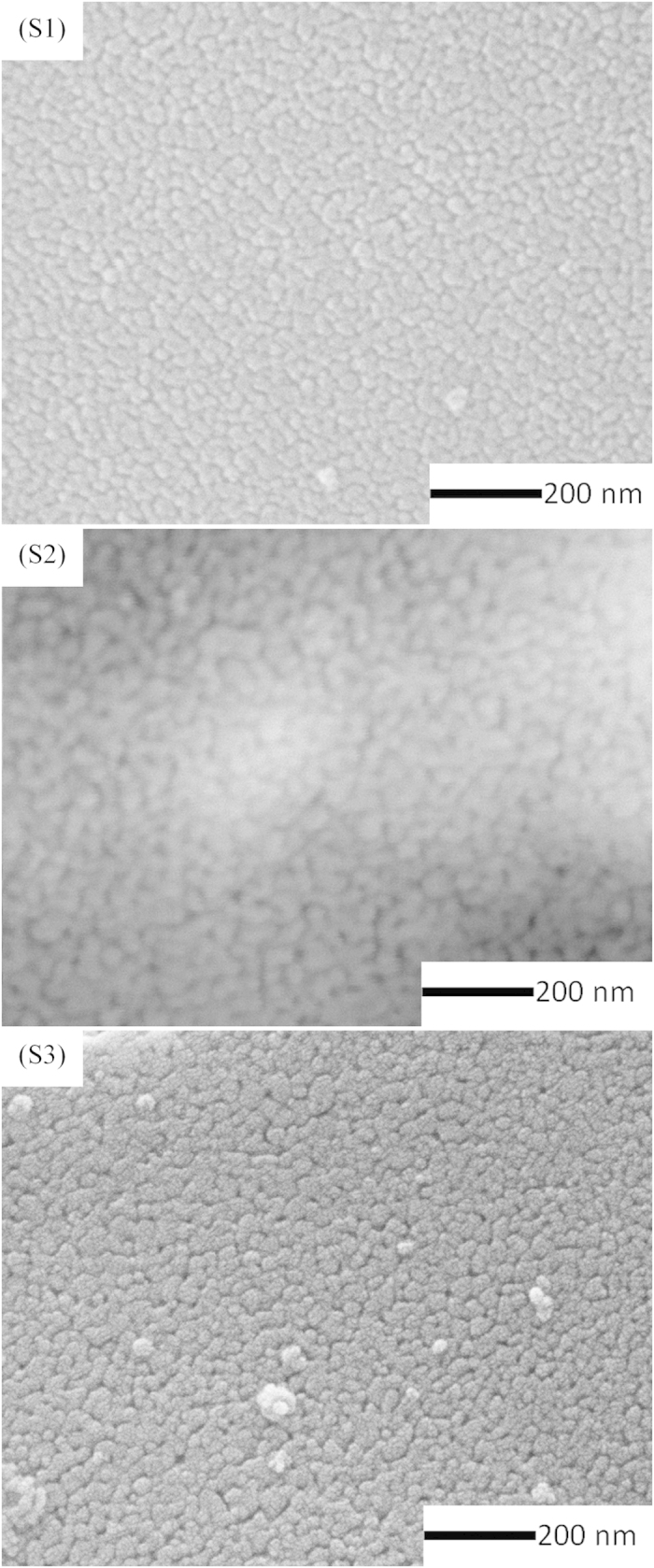
SEM images and particle size distribution of ZnAl_2_O_4_ xerogel prepared from (S1) Al_2_(SO_4_)_3_∙18H_2_O, (S2) AlCl_3_∙6H_2_O, and (S3) Al(NO_3_)_3_∙9H_2_O and sintered at 700 °C.

**Figure 6:**
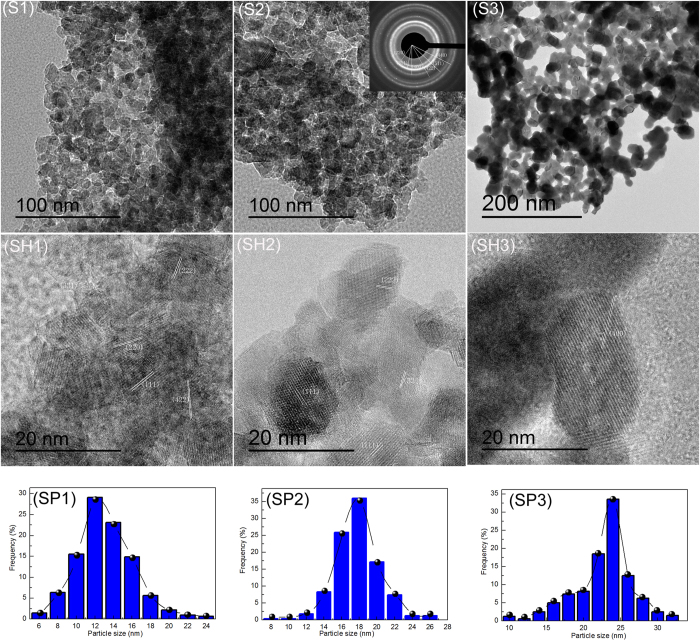
(S1–S3) TEM image, (SH1- SH3) HRTEM image, and (SP1–SP3) Particle size distribution of ZnAl_2_O_4_ nanoparticles prepared from (S1) Al_2_(SO_4_)_3_∙18H_2_O, (S2) AlCl_3_∙6H_2_O, and (S3) Al(NO_3_)_3_∙9H_2_O and sintered at 700 °C. The inset presents the SAED pattern taken from a portion of the ZnAl_2_O_4_ nanoparticles shown in Fig. 6(S2).

In order to investigate the effects of different aluminum salts on the formation of ZnAl_2_O_4_, including the particle size and surface morphology, SEM and TEM images were collected of the ZnAl_2_O_4_ samples prepared using different aluminum salts and sintered at 700 °C, and are shown in Fig. 5 and Fig. 6. The SEM images of the ZnAl_2_O_4_ samples reveal that the particles are almost spherical in shape and have a narrow particle size distribution (Fig. 5(S1–S3)). When AlCl_3_∙6H_2_O is added into the ZnAl_2_O_4_ precursor, large particles form, however, if Al_2_(SO_4_)_3_∙18H_2_O is added into the precursor, small particles with little adhesion are observed, as shown in Fig. 5(S1-S2).

Figure 6 shows (S1–S3) TEM image, (SH1- SH3) HRTEM image, and (SP1–SP3) the particle size distribution of ZnAl_2_O_4_ nanoparticles prepared from (S1) Al_2_(SO_4_)_3_∙18H_2_O, (S2) AlCl_3_∙6H_2_O, and Al(NO_3_)_3_∙9H_2_O. The ZnAl_2_O_4_ nanoparticles are spherical in shape with a narrow particle size distribution, as shown in Fig. 6(S1–S3). The corresponding particle size distribution patterns are given in the Fig. 6 (SP1–SP3). The average particle sizes of samples S1, S2, and S3 are around 12, 18, and 24 nm, respectively. TEM results show that the particle size variation tendencies for samples S1, S2, and S3 are consistent with those calculated from XRD patterns (Fig. 1). In addition, the BET surface area of the sample decreases with the increase of particle size as shown in Table 1. Compared with sample S2, the average particle size and BET surface area of sample S1 is significantly reduced, an effect which may be due to the SO_4_^2 −^ anion forming a bridged bidentate structure^31^. Fig. 6(S2) inset presents the SAED pattern taken from a portion of the ZnAl_2_O_4_ nanoparticles shown in Fig. 6(S2). The associated electron diffraction pattern is consistent with that of pure ZnAl_2_O_4_ crystals of a spinel structure, indexed as shown in Fig. 1(a). The SAED pattern revealed that the ZnAl_2_O_4_ nanoparticles possess interplanar spacings of 2.8235, 2.4156, 2.0205, 1.6328, 1.5531, and 1.4203 Å corresponding to the (220), (311), (400), (422), (511), and (440) planes, respectively.

Figure 6(SH1)-(SH3) shows the HRTEM image of ZnAl_2_O_4_ nanoparticles prepared from (S1) Al_2_(SO_4_)_3_∙18H_2_O, (S2) AlCl_3_∙6H_2_O, and (S3) Al(NO_3_)_3_∙9H_2_O. For these samples, the lattice planes of ZnAl_2_O_4_ nanoparticles were (111) with a lattice space of 4.6687 Å, (220) with a lattice space of 2.8437 Å, (222) with a lattice space of 2.3345 Å, (400) with a lattice space of 2.0210 Å, (331) with a lattice space of 1.8550 Å, and (440) with a lattice space of 1.4356 Å. It has been noted that (111), (220), (222), (400), (331) and (440) planes can be attributed to spinel ZnAl_2_O_4_ structure corresponding to JCPDS card No. 05–0669.

**Figure 7:**
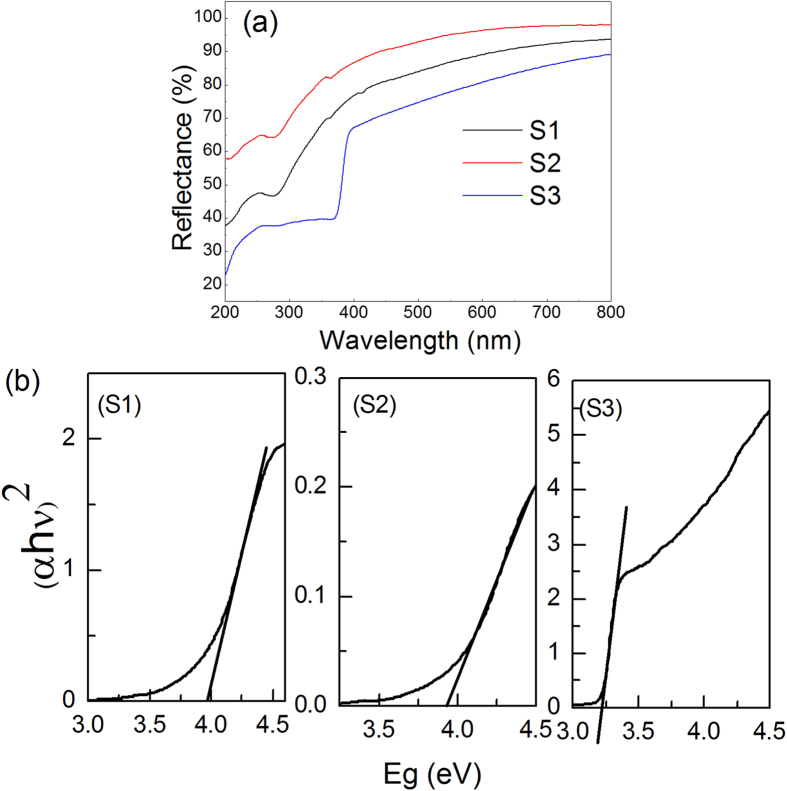
(**a**) UV-Vis diffuse reflectance spectra and (b) Eg values of ZnAl_2_O_4_ samples prepared from (S1) Al_2_(SO_4_)_3_∙18H_2_O, (S2) AlCl_3_∙6H_2_O, and (S3) Al(NO_3_)_3_∙9H_2_O and sintered at 700 °C.

Figure 7(a) shows the UV-Vis diffuse reflectance spectra of ZnAl_2_O_4_ samples prepared from (S1) Al_2_(SO_4_)_3_∙18H_2_O, (S2) AlCl_3_∙6H_2_O, and (S3) Al(NO_3_)_3_∙9H_2_O. Considering that ZnAl_2_O_4_ is a direct bandgap semiconductor^32^, the Eg of the ZnAl_2_O_4_ samples can be estimated from plots of (αhν)^2^ versus hν using the Tauc relation^33^. This is shown in Fig. 7(b), where h is the Planck constant, α is the Kubelka–Munk (K–M) absorption coefficient, and ν is the frequency. The linear portions of the plots are extrapolated to the hν axis to give the values of Eg. The calculated Eg values of samples S1, S2, and S3 are 3.98 ± 0.01, 3.92 ± 0.01 and 3.22 ± 0.01 eV, respectively. Generally, the Eg values of nano-sized semiconductors increase with a decrease in particle size. In this case, the observed phenomenon is consistent with what has been previously reported.

**Figure 8:**
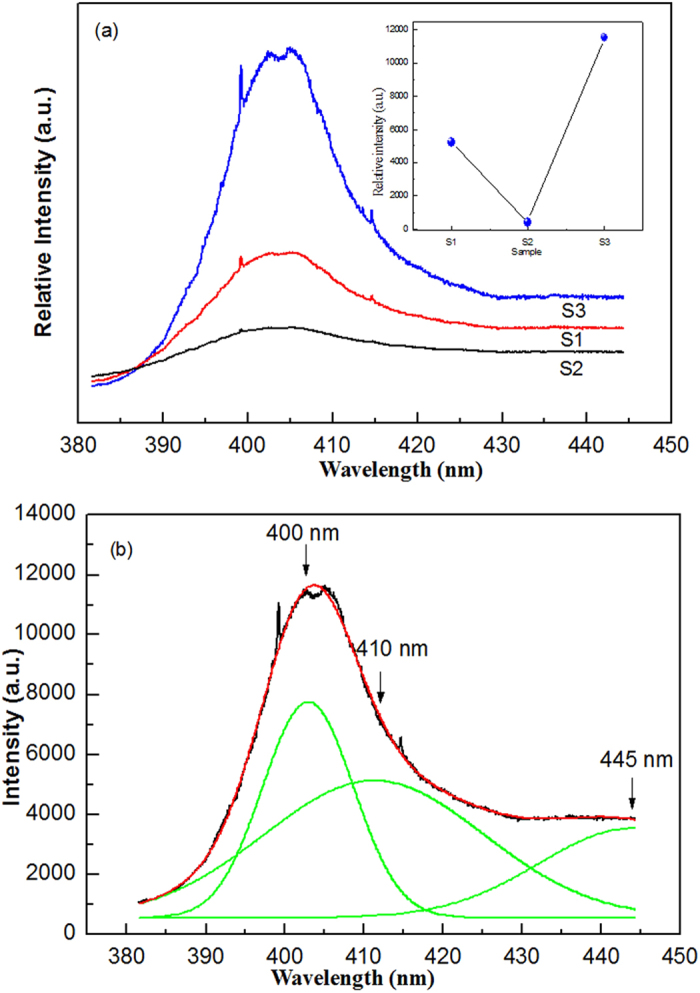
Fluorescence spectra of ZnAl_2_O_4_ samples prepared from (S1)Al_2_(SO_4_)_3_∙18H_2_O, (S2) AlCl_3_∙6H_2_O, and (S3) Al(NO_3_)_3_∙9H_2_O and sintered at 700 °C.

Figure 8(a) show the fluorescence spectra of ZnAl_2_O_4_ samples prepared from (S1) Al_2_(SO_4_)_3_∙18H_2_O, (S2) AlCl_3_∙6H_2_O, and (S3) Al(NO_3_)_3_∙9H_2_O and sintered at 700 °C. The fluorescence spectra with a wavelength range of 385 ~ 445 nm for all samples are presented after excitation with light, *λ*_*exc*_ = *325 *nm. The fluorescence spectrum of the ZnAl_2_O_4_ sample is wide and can be resolved using three Gaussian peaks at 400, 410, and 445 nm (Fig. 8b). The three emission peaks at 400, 410, and 445 nm can be ascribed to intra band gap defects such as oxygen vacancies^34^. It has been noted that there is an obvious increase in the fluorescence intensity of the peak located at 400 nm when the Eg value decreases (see Fig. 8(a) inset). Compared with the ZnAl_2_O_4_ nanoparticles prepared from Al_2_(SO_4_)_3_∙18H_2_O, the ZnAl_2_O_4_ nanoparticles obtained from AlCl_3_∙6H_2_O have a smaller Eg value, however, their fluorescence intensity is also smaller. Therefore, in this case we cannot simply attribute the differences in the fluorescence intensity for the three ZnAl_2_O_4_ samples only to the differences in their Eg values. The difference in the fluorescence intensity of the two ZnAl_2_O_4_ samples may also be related to the different phase purities of ZnAl_2_O_4_ samples prepared from (S1) Al_2_(SO_4_)_3_∙18H_2_O and (S2) AlCl_3_∙6H_2_O. In the case of the ZnAl_2_O_4_ sample prepared from Al_2_(SO_4_)_3_∙18H_2_O, traces of the S=O and S-O are visible in the FT-IR spectrum after sintering at 700 °C.

## Discussion

**Figure 9:**
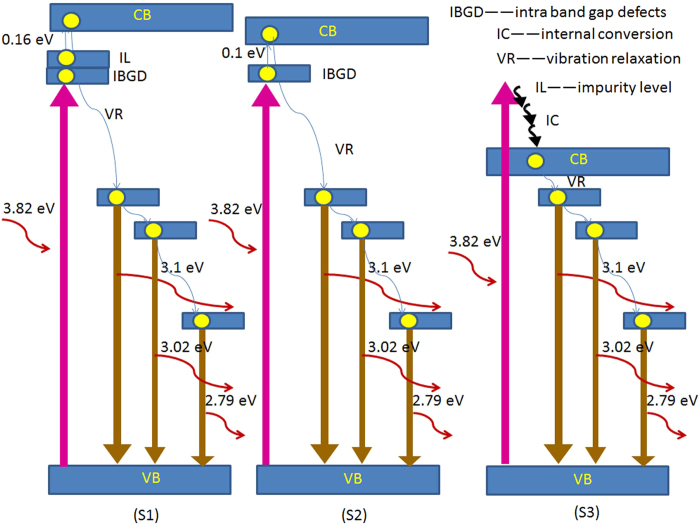
Fluorescence mechanism of ZnAl_2_O_4_ samples prepared from (S1) Al_2_(SO_4_)_3_∙18H_2_O, (S2) AlCl_3_∙6H_2_O, and (S3) Al(NO_3_)_3_∙9H_2_O.

In order to understand the fluorescence mechanism of the prepared ZnAl_2_O_4_ samples, it is necessary to propose a schematic band diagram to illustrate the process of excitation and emission for the system. Figure 9 shows a schematic band diagram for the fluorescence mechanism of ZnAl_2_O_4_ samples obtained from (S1) Al_2_(SO_4_)_3_∙18H_2_O, (S2) AlCl_3_∙6H_2_O, and (S3) Al(NO_3_)_3_∙9H_2_O. It is known that Al^3+^ 2*p* orbitals and *s* orbitals located at the upper part of the Al^3+^ 2*p* orbitals make up the major conduction band (CB) edge of ZnAl_2_O_4_, and the hybridization band composed of O^2−^ 2*p* and Zn^2+^ *3d* orbitals makes up the upper valence band (VB)^35^. When the Eg value >*λ*_*exc*_, (3.82 eV vs. 325 nm) it can be seen that one electron transition occurs from VB onto the intra band gap defects (IBGD) energy level (Fig. 9(S1) and (S2)). After that, the electron will be driven by continued transition from IBGD to CB. Then, the electron on the CB drops down to the low energy level through loss of energy by vibration relaxation (VR). Finally, the electron on the low energy level undergoes a radiative recombination with a hole in the valence band, accompanied by three blue-light emissions. For sample S1, the impurity (SO_4_^2−^) plays a crucial role to promote the electron transition from IL (impurity level) to CB and improve the fluorescence properties. When Eg < *λ*_*exc*_, one electron transition occurs from the VB to the high energy level (Fig. 9(S3)). Then, the electron on the high energy level drops down to the CB by internal conversion. At the same time, the electron on the CB by VR drops down to the low energy level with an accompanying loss of energy. Finally, the electron on the low energy level undergoes a radiative recombination with a hole in the valence band, accompanied by a series of blue-light emissions.

**Figure 10:**
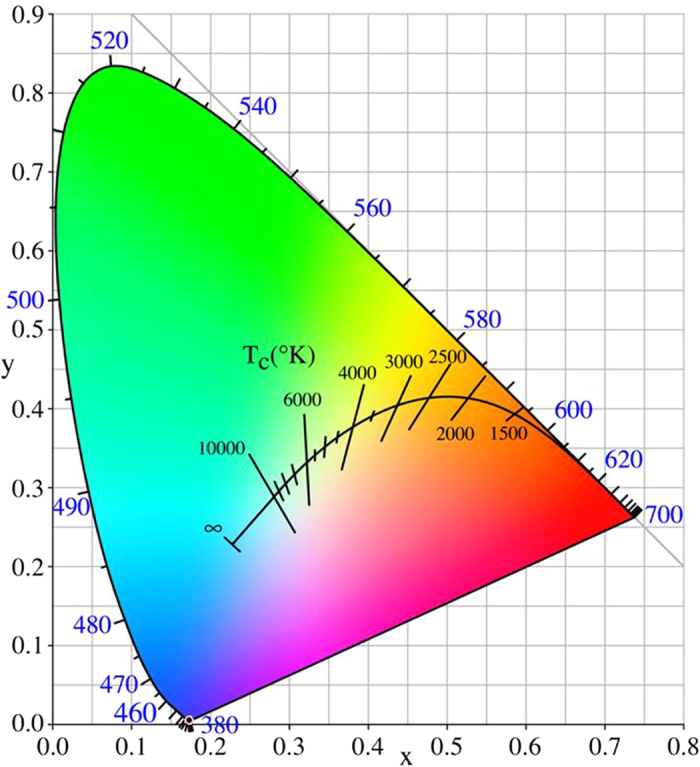
CIE diagram of ZnAl_2_O_4_ phosphor under 325 nm laser excitations.

Figure 10 shows the Commission International De I’Eclairage (CIE) diagram of a ZnAl_2_O_4_ phosphor under 325 nm laser excitations. The CIE color coordinates (x, y) of the ZnAl_2_O_4_ phosphor was calculated using the fluorescence spectra. A typical CIE color coordinate of a ZnAl_2_O_4_ phosphor was found to be x, y equals 0.1729, 0.0048 respectively under 325 nm laser excitations.

According to equation (7)^36^, the fluorescence quantum efficiency of the ZnAl_2_O_4_ phosphor can be calculated from the experimental data.


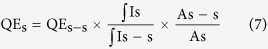


Where QE_s_ and QE_s-s_ are the fluorescence quantum efficiencies of the prepared sample and standard sample, respectively, under an excitation wavelength of 325 nm. ∫I_s_ and ∫I_s-s_ are the integrated emission intensities of the prepared sample and standard sample under an excitation wavelength of 325 nm. A_s_ and A_s-s_ are the absorbance of the prepared sample and standard sample under an excitation wavelength of 325 nm. The standard sample is 5-sulfosalicylic acid, which has a quantum efficiency of about 58% under an excitation wavelength of 325 nm. The fluorescence quantum efficiencies of samples S1, S2, and S3 were calculated to be 6.38%, 4.24%, and 10.11%, respectively. Observations made during these experiments show that the spinel ZnAl_2_O_4_ phosphor could be useful for blue light-emitting materials.

In summary, ZnAl_2_O_4_ nanoparticles with different particle sizes were prepared using a modified polyacrylamide gel route from different aluminum salt starting materials: Al_2_(SO_4_)_3_∙18H_2_O, AlCl_3_∙6H_2_O, and Al(NO_3_)_3_∙9H_2_O. The type of aluminum salt used can markedly influence the phase purity, particle size, and optical and fluorescence properties of the final product. Under 325 nm excitation, samples of ZnAl_2_O_4_ phosphor show blue emissions and the CIE colour coordinate was found to be (0.1729, 0.0048). The fluorescence mechanisms of the ZnAl_2_O_4_ phosphor have been discussed based on the experimental results. The fluorescence experiments revealed that the as-prepared ZnAl_2_O_4_ phosphor exhibits interesting abilities for application in blue light-emitting materials. Interestingly, similar preparation methods may be employed for the synthesis of other metal oxides nanoparticles, including fluorescence materials, multiferroic materials, oxide thermoelectric materials, photocatalytic materials, solid oxide fuel cell materials, and high-temperature superconducting materials.

## Methods

### Materials

Zn(NO_3_)_2_∙6H_2_O, AlCl_3_∙6H_2_O, Al_2_(SO_4_)_3_∙18H_2_O, and Al(NO_3_)_3_∙9H_2_O were purchased from DaMao Chem. Ltd., Tianjing. Citric acid (C_6_H_8_O_7_, AR), glucose (C_6_H_12_O_6._H_2_O, 99%), acrylamide (C_3_H_5_NO, AR), and N, N’-methylene-bisacrylamide (C_7_H_10_N_2_O_2_, 99%) were purchased from Kemiou Chem. Ltd., Tianjing and used without further purification.

### Synthesis

A certain stoichiometric amounts of Zn(NO_3_)_2_∙6H_2_O and different aluminum salts (Al_2_(SO_4_)_3_∙18H_2_O, AlCl_3_∙6H_2_O, and Al(NO_3_)_3_∙9H_2_O are labeled sample S1, S2 and S3, respectively) were dissolved in the deionized water to obtain a final solution of 0.015 mol/L with the total cations. Starting compositions of samples S1, S2 and S3 are given in Table 2. After the solution was transparent, a stoichiometric amount of chelating agent (citric acid) was added into the solution in the mole ratio 1.5:1 with respect to the total cations (*Zn*^*2+*^ and *Al*^*3+*^) to complex the cations. After that, 20 g glucose was dissolved into the solution. Finally, the acrylamide and N, N’-methylene-bisacrylamide monomers were added into the solution. The resultant solution was heated to 90 °C on a hot plate to initiate the polymerization reaction, and a few minutes later a polyacrylamide gel was formed. The gel was dried at 120 °C for 24 h in a thermostat drier. The obtained xerogel precursor was ground into powder and some powder was sintered at 600, 700, 800, and 900 °C for 5 h in air to prepare the objective products.

### Sample characterization

The ZnAl_2_O_4_ xerogel precursor sintered at 600 and 700 °C were analyzed by X-ray diffractometer (DX-2700) with Cu Kα radiation. Fourier transform infrared (FTIR) spectra in the range 400–2000 cm^−1^ were recorded using a Bruker IFS 66 v/S spectrometer. Thermogravimetric (TG) and differential thermal analysis (DTA) analyses were performed in a SDT Q600 (TA instruments, Inc. USA) simultaneous thermal analyzer at a heating rate of 10 °C/min. The surface morphology of the synthesized ZnAl_2_O_4_ sample was characterized by scanning electron microscopy (SEM) and transmission electron microscopy (TEM). The surface area of the samples were characterized by a 3H-2000BET-M instrument. The absorption spectra of the samples were examined on a Shimadzu UV-2500 UV-Visible spectrophotometer. The fluorescence spectra were collected at room temperature in a confocal Raman system using a He-Cd laser (325 nm, RGB laser system, NovaPro 30 mW, Germany).

## Additional Information

**How to cite this article**: Wang, S.-F. *et al.* A comparative study of ZnAl_2_O_4_ nanoparticles synthesized from different aluminum salts for use as fluorescence materials. *Sci. Rep.* **5**, 12849; doi: 10.1038/srep12849 (2015).

## Figures and Tables

**Table 1:** Crystallite size, particle size, Eg and BET specific surface area of samples S1, S2 and S3.

Sample	Crystallite size (nm)	Particle size (nm)	Eg (eV)	BET specific surface area (m^2^/g)
S1	13	12	3.98	40.13
S2	16	18	3.92	27.75
S3	24	24	3.22	19.38

**Table 2:** Starting compositions of samples S1, S2 and S3.

Sample	Al_2_(SO_4_)_3_∙18H_2_O	AlCl_3_∙6H_2_O	Al(NO_3_)_3_∙9H_2_O	Zn(NO_3_)_2_∙6H_2_O	citric acid	glucose	acrylamide	Bis-acrylamide
S1	6.6644g	/	/	1.4875 g	4.7282g	20 g	9.5958g	1.9192 g
S2	/	2.4143g	/	1.4875 g	4.7282g	20 g	9.5958g	1.9192 g
S3	/	/	3.7513g	1.4875 g	4.7282g	20 g	9.5958g	1.9192 g
